# Supplementary material for: Cytoglobin Silencing Promotes Melanoma Malignancy but Sensitizes for Ferroptosis and Pyroptosis Therapy Response
Source: Antioxidants (Basel). 2022 Aug 10;11(8):1548. doi: 10.3390/antiox11081548 (PMC9405091; doi:10.3390/antiox11081548)
Supplement: Supplementary file 1 [file antioxidants-11-01548-s001.zip › antioxidants-1842961-supplementary.pdf]

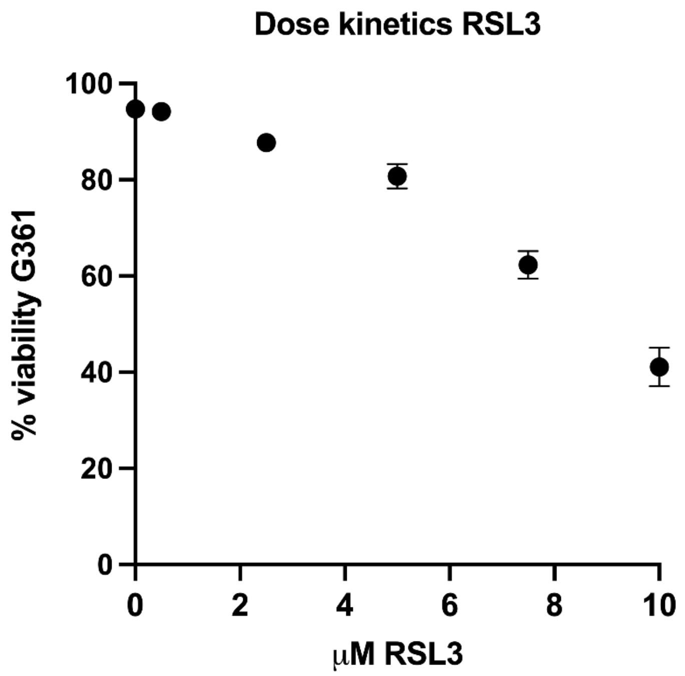

Figure S1. Dose titration RSL3. G361 cells were treated with different concentrations (0, 0.5, 2.5, 5, 7.5, 10 μM) of RSL3. Following propidium iodide (PI) staining, viability was measured 24 hours after treatment on the CytoFLEX flow cytometer.

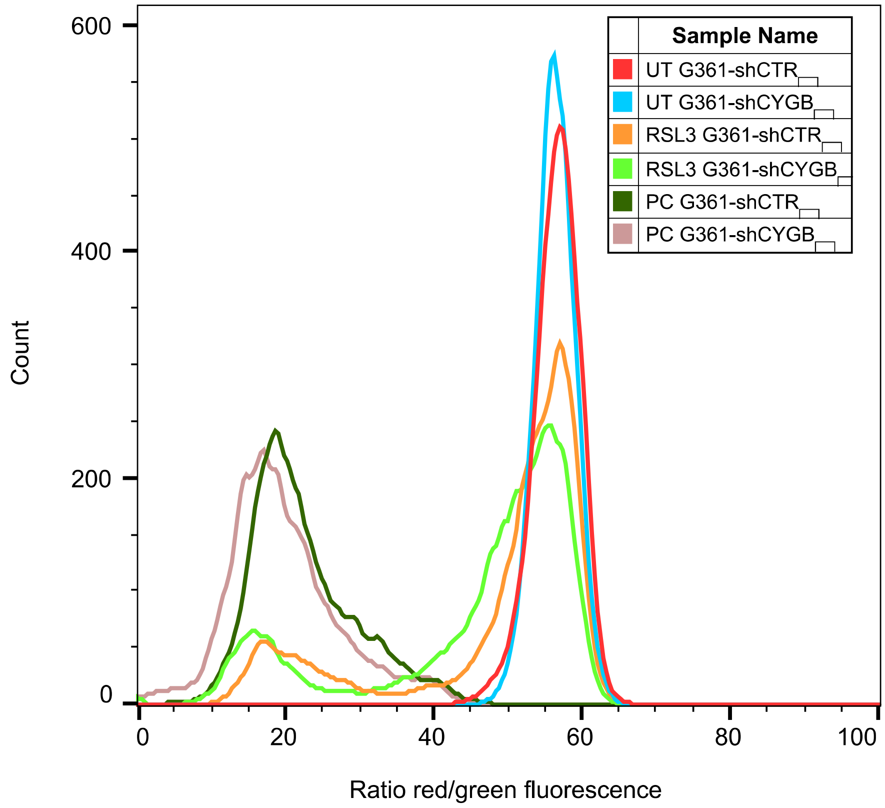

Figure S2. Lipid peroxidation in G361 cells. Lipid peroxidation measurements with Image-iT Lipid Peroxidation kit using flow cytometry. Lipid peroxidation Reagent is a ratiometric probe and the signal is detected on a flow cytometer with 488 nm laser excitation and fluorescence emission measured at 530/30 nm, and 532 nm laser excitation and fluorescence emission measured at 585/42 nm. The data are represented as the ratio of red/green fluorescence intensities. Ratios are higher in

untreated (UT) cell populations and upon treatment with 7.5 μM RSL3 and cumene hydroperoxide (positive control; PC). A decrease in red/green fluorescence intensity ratios is visible because of the increase in green signal as a result of cumene hydroperoxide and RSL3 induced lipid peroxidation in G361-shCTR and G361-shCYGB cells.

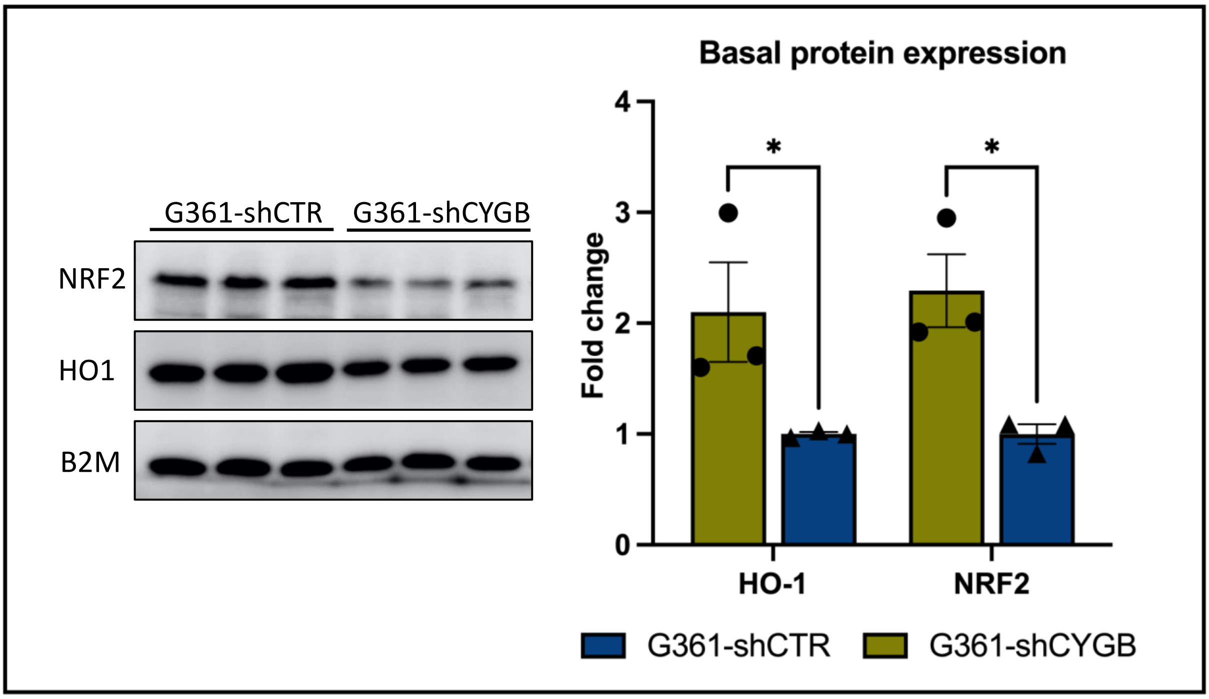

Figure S3. Basal protein expression. The protein expression of NRF2 and HO-1 was measured in G361-shCTR cells and G361-shCYGB melanoma cells. Quantification of the immunoblot signal showed that the knockdown of CYGB resulted in the decreased expression of NRF2 and HO-1. B2M was used as a loading control. Quantification is depicted as fold change compared to G361-shCYGB.Results are depicted as the mean with S.E.M of three independent experiments (n=3). Student's t-test (*$p \leq 0.05$).

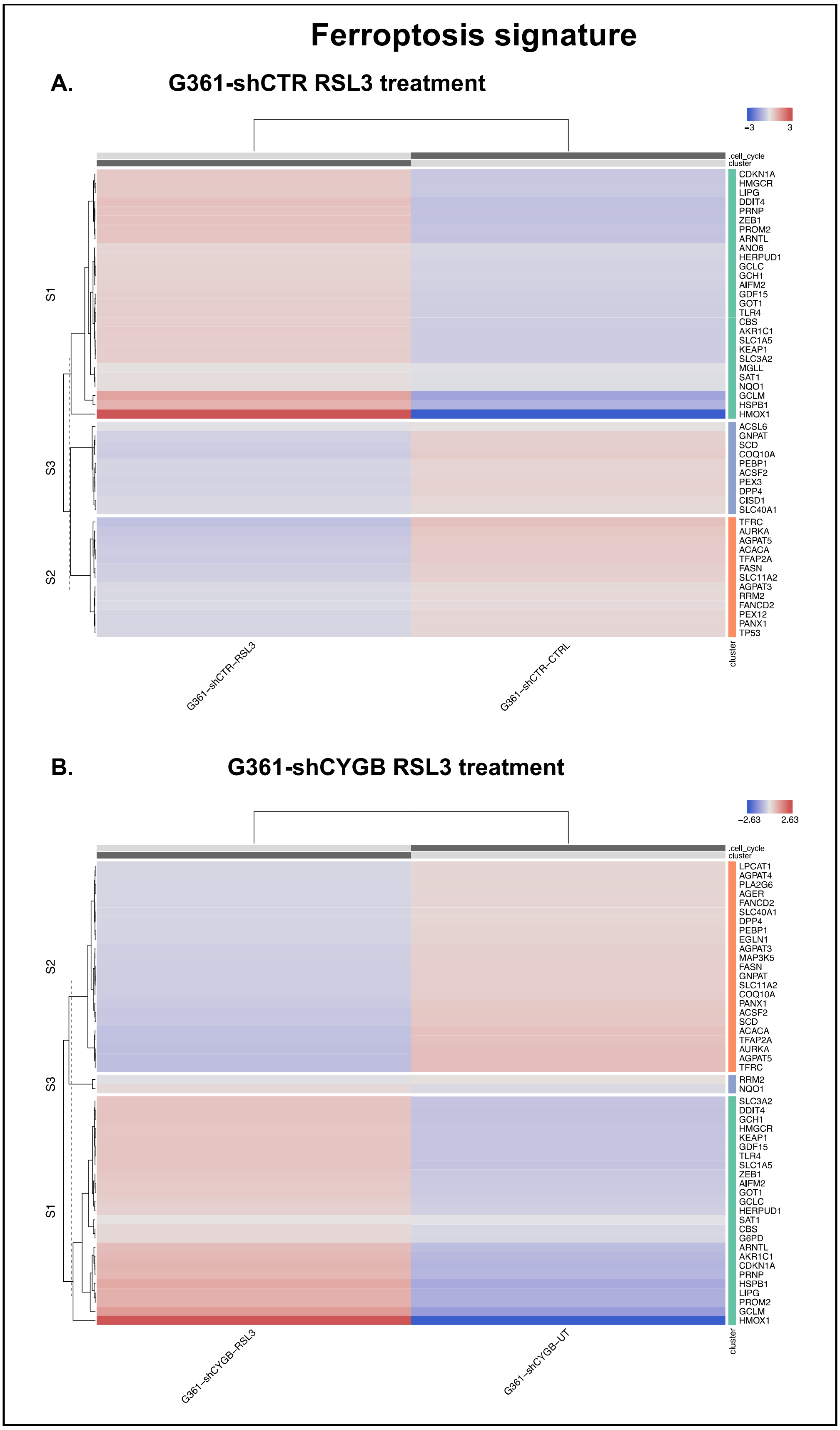

Figure S4. Ferroptosis signature heatmap. Hierarchical clustering of ferroptosis-related gene expression in (A) G361-shCTR and (B) G361-shCYGB upon RSL3 treatment. The top 50 ranked genes are represented.

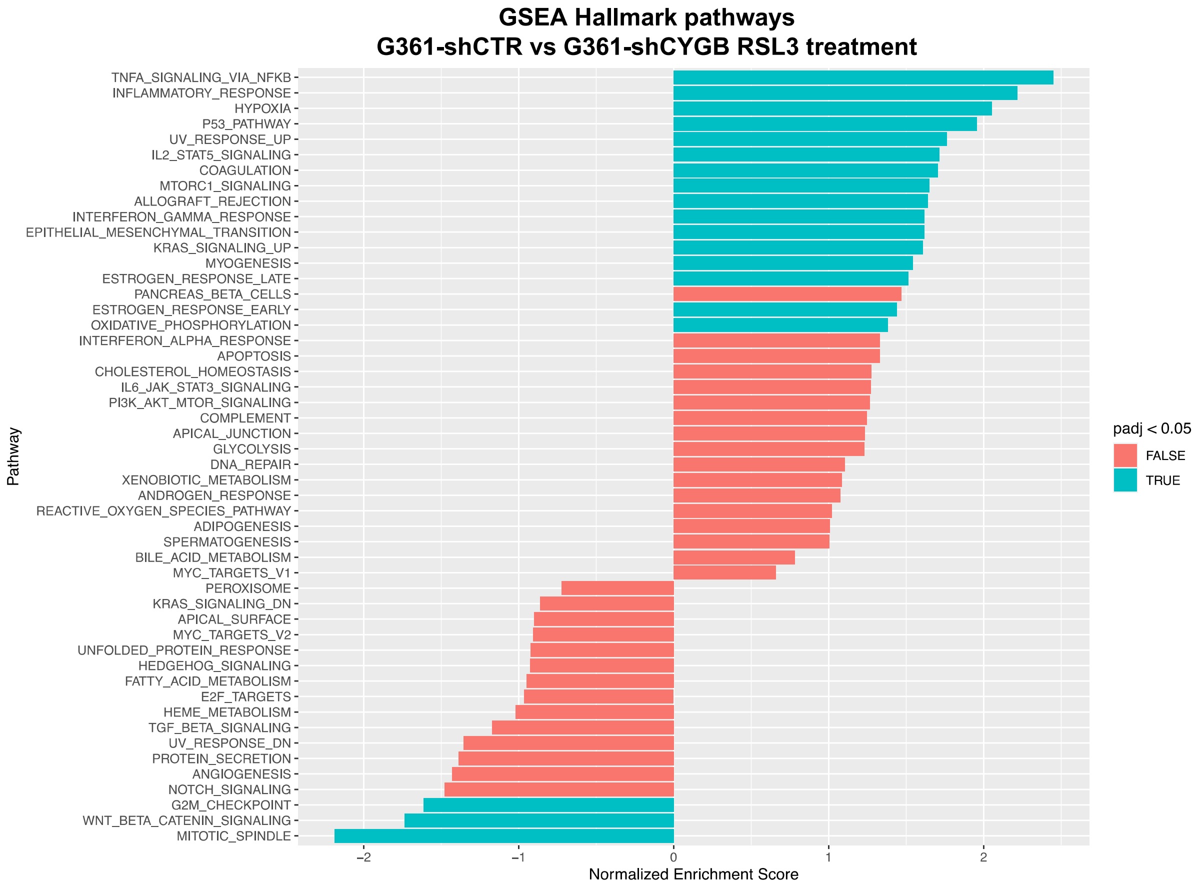

Figure S5. Gene Set Enrichment Analysis. Fast Gene Set Enrichment Analysis (fGSEA) using the hallmark pathway gene sets was performed on RSL3 treated G361-shCTR and G361-shCYGB melanoma cells.

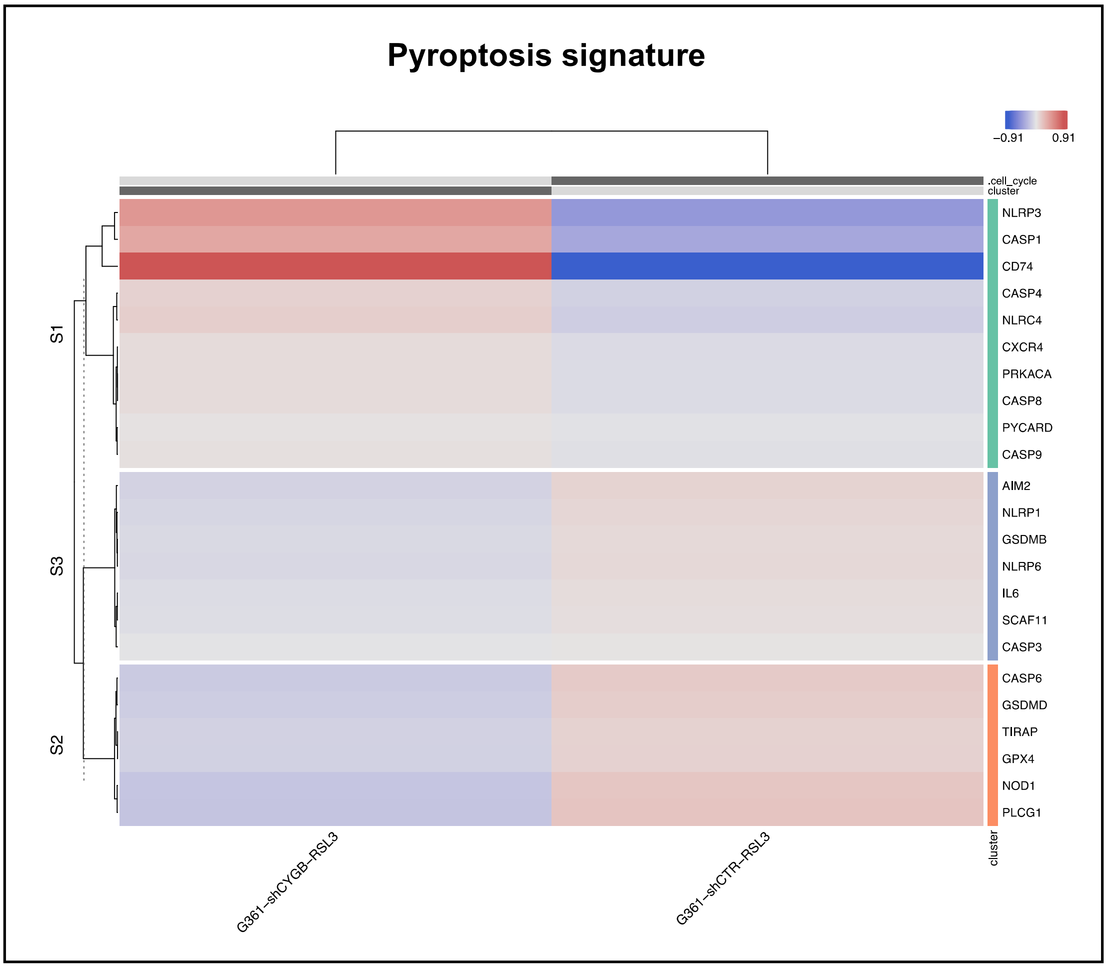

Figure S6. Pyroptosis signature heatmap. Hierarchical clustering of pyroptosis-related gene expression differences between RSL3 treated G361-shCTR and G361-shCYGB.

Table S1. Primer sequences of used genes

Gene symbol	Forward primer (5′ to 3′)	Reverse primer (5′ to 3′)
**Reference gene**		
B2M	TGCTGTCTCCATGTTTGATGTATCT	TCTCTGCTCCCCACCTCTAAGT
YWHAZ	ACTTTTGGTACATTGTGGCTTCAA	CCGCCAGGACAAACCAGTAT
**Target gene**		
CXCR4	ACTACACCGAGGAAATGGGCT	CCCACAATGCCAGTTAAGAAGA
CD74	GACGAGAACGGCAACTATCTG	GTTGGGGAAGACACACCAGC
CASP1	TTTCCGCAAGGTTCGATTTTCA	GGCATCTGCGCTCTACCATC
SYK	TGCACTATCGCATCGACAAAG	CATTTCCCTGTGTGCCGATTT
NLRP3	GATCTTCGCTGCGATCAACAG	CGTGCATTATCTGAACCCCAC
